# Proteinase Activated Receptors Mediate the Trypsin-Induced Ca^2 +^ Signaling in Human Uterine Epithelial Cells

**DOI:** 10.3389/fcell.2021.709902

**Published:** 2021-08-09

**Authors:** Anatoliy Shmygol, Jan J. Brosens

**Affiliations:** ^1^Department of Physiology, College of Medicine and Health Sciences, United Arab Emirates University, Al Ain, United Arab Emirates; ^2^Biomedical Sciences, Warwick Medical School, University of Warwick, Coventry, United Kingdom; ^3^Tommy’s National Miscarriage Research Centre, University Hospitals Coventry and Warwickshire NHS Trust, Coventry, United Kingdom

**Keywords:** proteinase-activated receptors, calcium signaling, endoplasmic reticulum, trypsin, endometrium, ENaC, Ishikawa cells

## Abstract

Embryo implantation is a complex and tightly regulated process. In humans, uterine luminal epithelium functions as a biosensor gauging the embryo quality and transmitting this information to the underlying endometrial stromal cells. This quality control ensures that only high quality embryos are implanted, while aberrant ones are rejected. The mechanisms of the embryo-uterine mucosa crosstalk remain incompletely understood. Trypsin, a serine protease secreted by the blastocyst, has been implicated in the cross-signaling. Here we address the mechanisms by which trypsin triggers the intracellular calcium signaling in uterine epithelium. We found that protease-activated G-protein coupled receptors are the main mechanism mediating the effects of trypsin in human uterine epithelium. In addition, trypsin activates the epithelial sodium channels thus increasing the intracellular Na^+^ concentration and promoting Ca^2+^ entry on the reverse mode of the sodium/calcium exchanger.

## Introduction

Pregnancy is a complex and highly coordinated physiological process that includes implantation, decidualization, placentation and birth of offspring through the process of parturition (Cha et al., 2012). The fertilized egg undergoes multiple mitotic divisions and enters the uterine cavity as a blastocyst. At the blastocyst stage, embryos mature, hatch from their outer shells (*zona pellucidae*) and then gain implantation competency. Further embryonic development critically depends on implantation of the hatched blastocyst into the maternal endometrium. The main purpose of implantation is to ensure that the blastocyst firmly anchors into the endometrial stroma, which will support the developing embryo and allow the process of placentation to begin (Wang and Dey, 2006). Implantation involves intimate interaction between an implantation-competent blastocyst and a receptive uterus, which occurs in a limited time period referred to as the window of implantation. Based on animal models, the uterine luminal epithelium has been considered solely responsible for the window of implantation. In polytocous species like mice or rats, transient expression of receptivity genes allow simultaneous implantation and correct spacing of multiple embryos at identical stages of development in an optimal endometrial environment (Kaneko et al., 2011). In contrast, humans, as a monotocous species, have to deal with highly invasive embryos that often display genomic instability and chromosomal aberrations (Mertzanidou et al., 2013). Under these conditions, the luminal epithelium functions as a biosensor gauging the embryo quality and transmitting this information to the underlying endometrial stromal cells to induce an environment that either supports further implantation or discards the aberrant embryo by menstruation-like shedding of the endometrium. Although a multitude of cellular signaling pathways involved in embryo-uterine crosstalk have been identified through gene expression studies and transgenic mouse models, a comprehensive understanding of the mechanisms of embryo-uterine interaction is still missing (Aplin, 2006; Zhang et al., 2013). Our previous work has shown that many metabolic and pro-inflammatory genes in human and murine endometrium are up-regulated in response to embryo-conditioned culture medium, suggesting that developing embryos secrete humoral factor(s) capable of inducing profound transcriptional responses in the endometrium (Brosens et al., 2014). Experiments in mice have shown that embryo-released serine protease, trypsin, triggers intracellular Ca^2+^ ([Ca^2+^]_i_) transients that lead to prostaglandin E_2_ (PGE_2_) release, phosphorylation of the transcription factor CREB and up-regulation of cyclooxygenase 2 (COX-2) required for prostaglandin synthesis from arachidonic acid (Ruan et al., 2012). This study has demonstrated the involvement of the epithelial Na^+^ channel (ENaC) in trypsin-induced [Ca^2+^]_i_ transients. Similarly to other epithelia (Rossier and Stutts, 2009), trypsin activates the uterine ENaC via proteolytic cleavage of the gamma subunit leading to inward current and membrane depolarization. The authors proposed that this depolarization triggers Ca^2+^ entry into epithelial cells through voltage-gated L-type Ca^2+^ channels (Ruan et al., 2012). However, evidence for the involvement of voltage-gated Ca^2+^ channels in uterine epithelial responses is not very convincing since trypsin-induced [Ca^2+^]_i_ transients were only partially inhibited by high doses (above 10 μM) of the L-type Ca^2+^ channel blocker nifedipine. At these concentrations, nifedipine may affect other ion channels/transporters in addition to the voltage-gated Ca^2+^ channel blockade. Indeed, a direct inhibition of alveolar ENaC by high doses of voltage-gated Ca^2+^ channel blockers has been described in the literature (Marunaka and Niisato, 2002). Most likely, mechanisms underlying the trypsin-induced Ca^2+^ signaling in uterine epithelium are more complex than a simple Ca^2+^ entry through voltage-gated Ca^2+^ channels. Nonetheless, the findings presented by Ruan et al. (2012) are important as they draw attention to a novel role for the embryo-derived serine proteases, namely their contribution to the embryo-uterine signaling dialogue, and highlight the importance of epithelial [Ca^2+^]_i_ signaling for embryo implantation. Previous studies have demonstrated that trophoblast spheroids can elevate [Ca^2+^]_i_ in RL95-2 cells, a human uterine epithelial cell line, by activating Ca^2+^ entry via mechano-sensitive Ca^2+^ permeable channels leading to the induction of epithelial adhesiveness (Tinel et al., 2000). However, the mechanism(s) mediating the protease-induced [Ca^2+^]_i_ signaling in human uterine epithelium remains incompletely understood. In this report, we present evidence for the protease-activated G-protein coupled receptors (GPCR) involvement in trypsin-induced Ca^2+^ signaling in the primary human uterine epithelium and in the Ishikawa cell line.

## Materials and Methods

### Cell Culture

Initial experiments were performed on primary cultures of human uterine epithelial cells. Endometrial biopsies were obtained from women attending the Implantation Research Clinic, University Hospitals Coventry and Warwickshire National Health Service Trust. Written informed consent was obtained in accordance with the Declaration of Helsinki 2000. The study was approved by the NHS National Research Ethics Committee of Hammersmith and Queen Charlotte’s Hospital NHS Trust (1997/5065). Endometrial biopsies were obtained from four women attending the Implantation Clinic at University Hospitals Coventry and Warwickshire NHS Trust. All research was undertaken with full ethical approval and with written informed consent obtained from all participants in accordance with The Declaration of Helsinki 2000. Biopsies were taken during the secretory phase of the menstrual cycle using an Endocell^TM^ cannula, starting from the uterine fundus and moving downward to the internal cervical ostium. The biopsy was collected in a labeled Bijoux tube containing 5 ml of cell culture medium and transferred to the lab for processing as quickly as possible. The endometrial epithelial cells were isolated from the endometrial biopsies using collagenase and DNase digestion as described in detail elsewhere (Barros et al., 2016). After separation of uterine glands from the stromal and immune cells, the former were further digested with trypsin to obtain a single-cell suspension. Dissociated epithelial cells were plated into glass-bottomed 35 mm Petri dishes (Mattek) and incubated at 37°C in a humidified incubator in 5% CO_2_ for up to a week before using them in Ca^2+^ imaging experiments. Three to six Petri dishes per biopsy were used in the Ca^2+^ imaging experiments.

In the initial experiments, we found that primary uterine epithelial cells generated [Ca^2+^]_i_ responses to trypsin application similar to those observed in Ishikawa cells, an uterine epithelial cancer cell line. Given this similarity between the primary human endometrial epithelial cells and the Ishikawa cell line, subsequent mechanistic experiments were performed on Ishikawa cells. The Ishikawa cell line was obtained from Sigma-Aldrich and cultured in glass-bottomed 35 mm Petri dishes using a cell culture method similar to that described by Schmidt and colleagues (Schmidt et al., 2014). Ishikawa cells were used in Ca^2+^ imaging experiments within a week after passaging. Before the experiment, the cells were cultured overnight in serum-free DMEM.

### Ca^2+^ Imaging

For imaging intracellular calcium ([Ca^2+^]_i_) responses to trypsin, a ratiometric Ca^2+^-sensitive indicator Fura-2 was employed. The cells were washed with physiological saline solution (PSS) of the following composition (in mmol/l): 135 NaCl, 4 KCl, 1.2 MgCl_2_, 2 CaCl_2_, 5 HEPES and 10 Glucose. The pH of this solution was adjusted to 7.4 with NaOH. The Fura-2 indicator was loaded into cells as acetoxymethyl ester dissolved in DMSO and diluted with PSS to a final concentration of 2.5 μM. The loading solution also contained 0.05% of non-ionic detergent Pluronic F127 to aid the dye dispersal and 2 mM probenecid to facilitate dye retention in the cytoplasm. After 60 min loading at room temperature the cells were washed with PSS and incubated on the bench while protected from ambient light for another 30 min to ensure complete de-esterification of the dye. After that, the Petri dish with Fura-2 loaded cells was mounted on the stage of an inverted microscope (Olympus IX71, United Kingdom) and superfused with pre-warmed to 35°C PSS. Trypsin and other drugs were applied to cells within the field of view using a gravity-fed multi-tube preheater (MPRE8, Cell MicroControls, United States). A fast monochromator (Polychrome V, TILL Photonics, Germany) provided Fura-2 excitation alternating between 360 ± 12 and 380 ± 12 nm. The resulting Fura-2 fluorescence was separated from the excitation light by a 495 nm dichromatic mirror (Chroma, United States) and recorded at a wavelength above 510 nm. Time series of fluorescence images were collected at 1 ratio-pair per second using a front-illuminated EMCCD camera (Luca, Andor, United Kingdom) controlled by the WinFluor software package (J. Dempster, Strathclyde University, United Kingdom). The camera exposure was set to 50 ms and the cells were not illuminated for the rest of the cycle time. This substantially reduced the photo-bleaching of Fura-2 during long-lasting experiments. The same software was used for the extraction of fluorescence intensities and calculation of Fura-2 ratio representing the [Ca^2+^]_i_ dynamics.

All solutions were prepared using analytical grade salts and deionized water. Trypsin was supplied by Worthington Biochemical Corporation, United States. DMSO, Pluronic F127 and Fura-2/AM were purchased from the Molecular Probes, United States. PAR agonist and antagonist peptides, amiloride, SN-6 and the store operated channel inhibitor GSK7975A were supplied by Tocris Bioscience, United Kingdom. All other reagents were purchased from Sigma, United Kingdom.

### Data Quantification and Analysis

Background fluorescence was calculated from areas devoid of cells and subtracted from each time series. To quantify the cellular [Ca^2+^]_i_ responses to trypsin and other agents, regions of interest (ROIs) were drawn around each cell within the field of view, the mean intensity values were extracted from the ROIs and the ratio between fluorescence intensities exited at 360 and 380 nm were calculated in WinFluor. The technical graphing and data analysis software package IgorPro 7.0.8.1 (Wavemetrics, United States) was used for statistical analyses and preparation of figures. The data were obtained from at least three different cultures. Where appropriate, statistical comparisons were made using unpaired *t*-test. When multiple comparisons were made, a 1-way ANOVA followed by Dunnet’s *post hoc* test was used.

## Results

**FIGURE 1:**
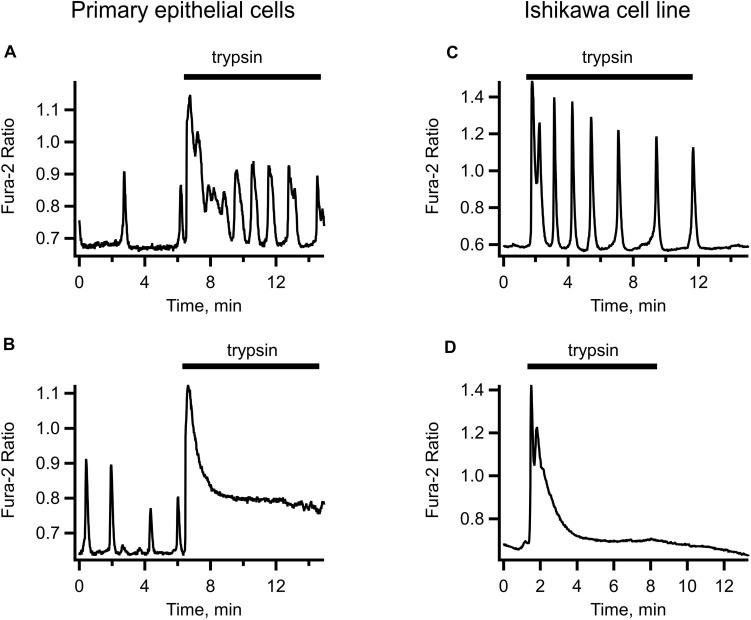
Examples of trypsin-induced [Ca^2+^]_i_ signals in human primary uterine epithelial cells (left-hand panel) and in the Ishikawa cell line (right-hand panel). Application of 5 nM trypsin (indicated by a solid bar above each trace) triggered an initial transient elevation of [Ca^2+^]_i_ followed by [Ca^2+^]_i_ oscillations of variable frequency and amplitude **(A,C)** or by a non-oscillatory plateau of variable height **(B,D)**. Records are typical of 87 cells from 4 separate cultures.

We reasoned that the concentrations of the embryo-secreted protease are unlikely to reach a micro-molar level around the uterine epithelium during human embryo implantation. We therefore tested a low-nanomolar concentration range of trypsin for its effectiveness at triggering the [Ca^2+^]_i_ responses in primary uterine epithelial cells and in the Ishikawa cell line. Figure 1 illustrates typical [Ca^2+^]_i_ responses to the application of 5 nM trypsin dissolved in PSS. In the majority of cells, trypsin at this concentration triggered a rapid transient rise in [Ca^2+^]_i_ followed by a long-lasting oscillation (Figures 1A,C). In about a quarter of all cells tested (31 out of 132), a steady elevation of [Ca^2+^]_i_ was observed after the initial [Ca^2+^]_i_ transient instead of oscillations. The responses to 1 and 0.5 nM trypsin were similar to that triggered by 5–10 nM but the number of cells responding to trypsin declined in proportion to the decrease in trypsin concentration (not illustrated). We elected to use 5–10 nM trypsin in all the subsequent experiments.

The [Ca^2+^]_i_ dynamics were very similar in the primary epithelium and in Ishikawa cells, bar for the occasional irregular spontaneous activity sometimes observed in primary epithelium before the application of trypsin but never in the cell line (compare left-hand and right-hand panels of Figure 1). Given the similarity of trypsin-induced [Ca^2+^]_i_ signaling in Ishikawa cells to that in primary uterine epithelium and the amenity of the cell line to different experimental maneuvers, we used Ishikawa cells to conduct in subsequent experiments.

### Store Operated Ca^2+^ Entry Is Vital for the Oscillations

**FIGURE 2:**
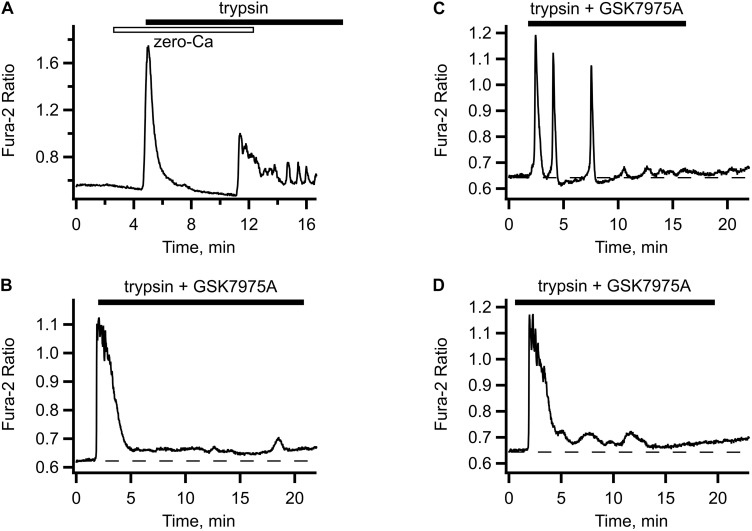
Store-operated Ca^2+^ entry contributes to trypsin- induced [Ca^2+^]_i_. **(A)** In Ca^2+^-free solution, trypsin application produced only the initial [Ca^2+^]_i_ transient but no oscillations (typical of *n* = 7). Upon re-addition of extracellular Ca^2+^, [Ca^2+^]_i_ oscillations reappeared. **(B–D)** Typical responses to trypsin (5 nM) in the presence of store operated Ca^2+^ channel inhibitor GSK7975A. Addition of GSK7975A (5 μM) to trypsin containing solution abolished the oscillations, either immediately **(B)** or after several oscillations of diminishing amplitude **(C)** leaving behind a slightly elevated level of [Ca^2+^]_i_ sometimes accompanied by slow irregular fluctuations **(D)** (*n* = 34 cells from two different cultures).

When trypsin was applied in the absence of extracellular Ca^2+^, there was only a single [Ca^2+^]_i_ transient observed, which was followed by an exponential decline below the pre-stimulation level. Readmission of extracellular Ca^2+^ in a continuous presence of trypsin triggered immediate rise in [Ca^2+^]_i_ and reappearance of the oscillations (illustrated in Figure 2A). To test the route for the extracellular Ca^2+^ entry, we employed GSK7975A (Derler et al., 2013), a specific inhibitor of the store operated Ca^2+^ channels. In the presence of this inhibitor, trypsin-induced [Ca^2+^]_i_ oscillations were abolished, either immediately (Figure 2B) or after several declining oscillations (Figure 2C). The initial [Ca^2+^]_i_ transient was unaffected by the GSK7975A application, but it was not followed by the oscillations, only a slightly elevated [Ca^2+^]_i_ level was maintained. Sometimes this was accompanied by slow and irregular fluctuations of [Ca^2+^]_i_ (Figure 2D). These results suggest that the main mechanism that sustains the trypsin-induced [Ca^2+^]_i_ oscillations was Ca^2+^ entry through the STIM/Orai store operated Ca^2+^ channels, a hallmark feature of the GPCR-mediated Ca^2+^ signaling. We explored this in the next series of experiments.

### Effects of PAR Peptides

**FIGURE 3:**
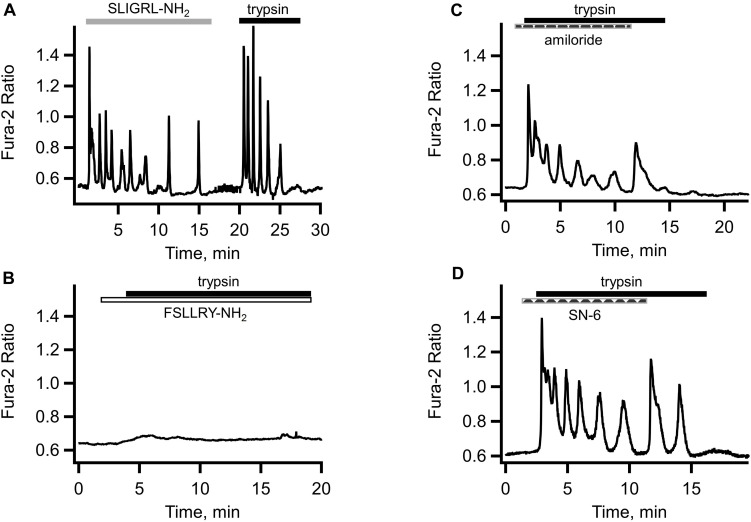
Role of proteinase activated receptors, ENaC and sodium/calcium exchanger in the trypsin-induced [Ca^2+^]_i_ responses. **(A)** Application of PAR2 agonist SLIGRL-NH_2_ (10 μM, empty bar) produced a [Ca^2+^]_i_ response similar to that elicited by subsequent trypsin (5 nM, solid bar) application to the same cell. **(B)** Both the initial transient and [Ca^2+^]_i_ oscillations in response to trypsin (5 nM, solid bar) were abolished by the PAR2 inhibiting peptide FSLLRY-NH_2_ (10 μM, empty bar). Traces shown are typical of 46 cells from three separate cultures. **(C)** Application of ENaC inhibitor amiloride (10 μM, dashed bar) partially inhibited [Ca^2+^]_i_ oscillations but not the initial [Ca^2+^]_i_ transient in response to trypsin (5 nM, solid bar) (*n* = 113 cells from four separate cultures). **(D)** Inhibitor of the reverse mode of sodium/calcium exchanger SN-6 (10 μM, dashed bar) had similar to amiloride effect (*n* = 24 cell from three separate cultures).

Figure 3A illustrates the effect of the PAR2 activating peptide SLIGRL-NH_2_ application to Ishikawa cell. The response was similar to that induced by trypsin and comprised an initial transient rise in [Ca^2+^]_i_ due to the release from the intracellular store followed by [Ca^2+^]_i_ oscillations. After the washout of PAR2 agonist peptide, the cells were capable of responding to trypsin (Figure 3A). Application of TFLLR-NH_2_, the PAR1 activating peptide, also induced [Ca^2+^]_i_ responses in Ishikawa cells (not illustrated). On the other hand, pretreatment of the cells with FSLLRY-NH_2_, a PAR-2 inhibiting peptide, abolished both components of the [Ca^2+^]_i_ response to trypsin. That is, not only the [Ca^2+^]_i_ oscillations were abolished but the initial [Ca^2+^]_i_ transient was eliminated as well (see Figure 3B). Interestingly, a small and slow rise in [Ca^2+^]_i_, similar to that observed in the experiments with GSK7975A remained in the presence of FSLLRY-NH_2_. This may indicate the presence of an additional trypsin-activated Ca^2+^ entry pathway unrelated to the GPCRs or store-operated Ca^2+^ channels. An obvious candidate for such a mechanism could be ENaC that has already been shown to contribute to embryo implantation in mice (Ruan et al., 2012). We explored this in Ishikawa cells using amiloride, a potent inhibitor of ENaC.

### Role of ENaC

As illustrated in Figure 3C, ENaC inhibition failed to eliminate the initial component of the trypsin-induced [Ca^2+^]_i_ response but reduced the amplitude and, to a lesser extent, the frequency of subsequent [Ca^2+^]_i_ oscillations. De-inhibition of ENaC by rapid removal of amiloride led to a transient resurgence of the trypsin-induced [Ca^2+^]_i_ oscillations amplitude (Figure 3C). This was consistently observed in all 78 cells from five different cultures. A similar effect was observed in the experiments with SN-6, a preferential inhibitor of the reverse mode of the sodium/calcium exchanger (Niu et al., 2007; Gandhi et al., 2013). Pre-incubation of cells with 10 μM of SN-6 reduced the amplitude and frequency of [Ca^2+^]_i_ oscillations without affecting the initial [Ca^2+^]_i_ transient in response to trypsin (Figure 3D). There was also a re-bounce effect upon a rapid washout of SN-6. In fact, the resurgence of the [Ca^2+^]_i_ oscillation was more pronounced compared to that after the amiloride withdrawal. Taken together, these results suggest that the additional pathway for the extracellular Ca^2+^ entry is the reverse mode of the sodium/calcium exchanger (NCX).

## Discussion

**FIGURE 4:**
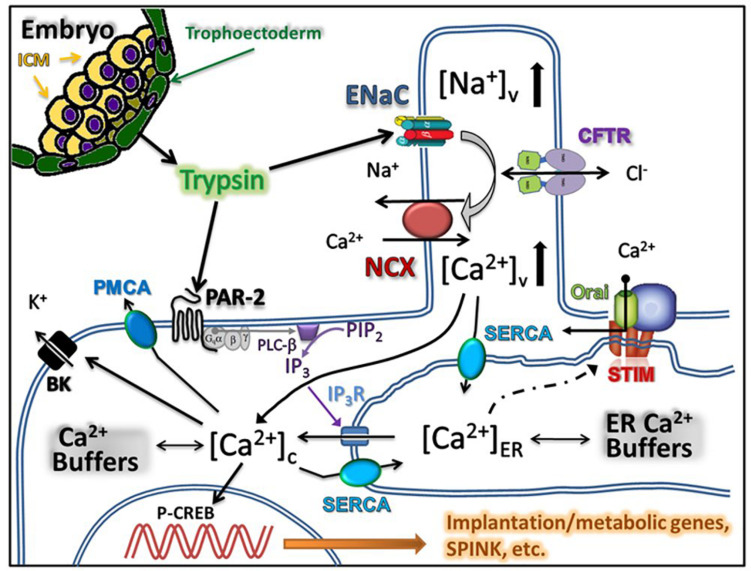
Summary of the proposed model for trypsin induced Ca^2+^ signaling in uterine epithelium. Trypsin is released by the trophectoderm of the implanting embryo and acts on proteinase activated receptors thereby triggering the release of Ca^2+^ from the endoplasmic reticulum. Emptying the reticulum triggers store-operated Ca^2+^ entry. In parallel, trypsin activates ENaC located in the microvilli of the uterine epithelial cells leading to the increase in the intravillous Na^2+^ level, reversal of the Na^+^/Ca^2+^ exchanger and additional Ca^2+^ entry into cytoplasm.

In this report, we examined the mechanism of the trypsin-induced [Ca^2+^]_i_ signaling in human uterine epithelial cells. Our main findings are: (i) the effects of trypsin application are similar in primary human uterine epithelium and in the Ishikawa cell line; and (ii) the peptide agonists of PAR1 and PAR2 can mimic the effects of low concentrations of trypsin on uterine epithelial [Ca^2+^]_i_ dynamics while the PAR inhibitor peptide eliminates this effect. These results are compatible with the idea that the proteinase activated receptors play a major role in the trypsin-induced endometrial [Ca^2+^]_i_ signaling. This is in contrast to the mechanism proposed for implantation in mice, where ENaC and the L-type Ca^2+^ channels are thought to be pivotal players (Ruan et al., 2012). Nevertheless, our results with ENaC inhibitor amiloride and the NCX inhibitor SN-6 suggest that ENaC-NCX system can provide an additional route for extracellular Ca^2+^ entry in parallel to that afforded by the PAR activation of store-operated Ca^2+^ channels. The schematic drawing in Figure 4 summarizes our understanding of the result obtained in this study. We suggest that trypsin released from the trophectoderm of implanting embryo binds to PAR2, thereby triggering a classical GPCR-mediated intracellular Ca^2+^ release followed by store-operated Ca^2^ entry via the STIM/orai voltage-independent Ca^2+^ channels. In parallel, trypsin cleaves the gamma-subunit of the epithelial sodium channel thereby activating the Na^+^ entry via ENaC. The role of epithelial sodium channels and the cystic fibrosis transmembrane conductance regulator (CFTR) chloride channels in uterine fluid secretion and reabsorption has long been recognized as an important determinant of uterine closure, a mechanism by which embryos are held in contact with the uterine epithelium before initiation of implantation (Chan et al., 2002; Naftalin et al., 2002). Similar to other ion transporting epithelia, uterine ENaC and CFTR co-localize to microvilli on the apical membrane and functionally interact with each other to provide electrically coupled ionic fluxes (Horisberger, 2003; Konstas et al., 2003). Inward currents through ENaC and CFTR are counterbalanced by outward current through the large conductance Ca^2+^-activated potassium channels (Zhang et al., 2012). We propose that Na^+^ entry into the intravillous space via trypsin-activated ENaC will depolarize the cellular membrane and increase the intravillous Na^2+^ concentration sufficiently high to reverse the NCX thereby providing means for Ca^2+^ entry into the intravillous space. Diffusion of Ca^2+^ from the microvilli into the bulk cytoplasm will increase [Ca^2+^]_i_ and, in parallel with store-operated calcium entry, act as a source for re-filling of the endoplasmic reticulum. Increased [Ca^2+^]_i_ will also activate the BK channels leading to repolarization and termination of Ca^2+^ entry via the reverse mode of NCX. Thus, the proposed two mechanisms will act in coherence to sustain the [Ca^2+^]_i_ oscillations. Misbalance between the rates of Ca^2+^ entry and Ca^2+^ accumulation into the endoplasmic reticulum may manifest itself as a steady, non-oscillatory Ca plateau of variable height, which we observed in a proportion of cells. The coexistence of the two parallel routes for Ca^2+^ entry will ensure a robust activation of prostaglandin E production vital for decidualization of endometrial stromal cells.

## Data Availability Statement

The raw data supporting the conclusions of this article will be made available by the authors, without undue reservation.

## Ethics Statement

The studies involving human participants were reviewed and approved by the NHS National Research Ethics Committee of Hammersmith and Queen Charlotte’s Hospital NHS Trust (1997/5065). The patients/participants provided their written informed consent to participate in this study.

## Author Contributions

AS and JB conceived and planned the study. JB collected endometrial biopsies and provided reagents and other materials. AS performed the experiments, analyzed the data, and wrote the manuscript. Both authors reviewed and approved the final version of the manuscript.

## Conflict of Interest

The authors declare that the research was conducted in the absence of any commercial or financial relationships that could be construed as a potential conflict of interest.

## Publisher’s Note

All claims expressed in this article are solely those of the authors and do not necessarily represent those of their affiliated organizations, or those of the publisher, the editors and the reviewers. Any product that may be evaluated in this article, or claim that may be made by its manufacturer, is not guaranteed or endorsed by the publisher.
